# Supplementary material for: A Practical Covariance-Based Method for Efficient Detection of Protein–Protein Attractive and Repulsive Interactions in Molecular Dynamics Simulations
Source: J Chem Inf Model. 2025 Sep 30;65(19):9865–70. doi: 10.1021/acs.jcim.5c01725 (PMC12529755; doi:10.1021/acs.jcim.5c01725)
Supplement: Supplementary file 1 [file ci5c01725_si_001.pdf]

Supporting Information

A Practical Covariance-Based Method for Efficient Detection of Protein-Protein Attractive and Repulsive Interactions in Molecular Dynamics Simulations

Mert Golcuk[1] and Mert Gur[1,*]

[1] Department of Computational and Systems Biology, School of Medicine, University of Pittsburgh, Pittsburgh, PA, 15213, USA

*Corresponding Author, gurmert@pitt.edu

Supporting Methods

Molecular Dynamics Simulation System Preparation.

In this study, we performed molecular dynamics (MD) simulations of the H11-H4 nanobody bound to the Omicron SARS-CoV-2 spike protein receptor-binding domain (RBD), following the same protocol as in our previous studies on the wild-type (WT) strain as well as the Alpha and Beta variant.[1,2] The crystal structure of the H11-H4 nanobody bound to the RBD at 1.85 Å resolution (PDB ID: 6ZBP[3]) was used as the starting template, and Omicron-specific mutations were introduced into the RBD using the 'Mutator Plugin' in VMD.[4] Each system was solvated with the TIP3P water model in a rectangular water box extending at least 25 Å beyond the protein complex in all directions, ensuring a minimum 50 Å surface-to-surface separation between the complex and its periodic images. Counterions were added to neutralize the system, and NaCl concentration was adjusted to 150 mM. The final solvated systems contained approximately 150,000 atoms. All system preparation steps were performed in VMD.

MD simulations were conducted under NPT ensemble conditions using NAMD versions 2.14 and 3.0,[5] the CHARMM36 force field,[6] and a 2 fs integration time step with all bonds involving hydrogen constrained using the SHAKE algorithm. Temperature was maintained at 310 K via Langevin dynamics with a damping coefficient of 1 ps^{-1}, and pressure was controlled at 1 atm using the Langevin Nosé–Hoover piston method with an oscillation period of 100 fs and a damping timescale of 50 fs. A 12 Å cutoff was applied for van der Waals interactions with a switching function between 10 and 12 Å, and long-range electrostatics were calculated using the particle-mesh Ewald method. Periodic boundary conditions were applied in all dimensions.

System equilibration followed a multistage protocol. First, each system was minimized for 10,000 steps and equilibrated for 2 ns with the protein fixed, allowing the solvent and ions to relax around the fixed protein complex. A second minimization-equilibration cycle was then performed: the full system was minimized for an additional 10,000 steps without restraints, followed by 4 ns of equilibration with harmonic constraints applied to C_α atoms. Finally, all restraints were removed, and the systems were equilibrated for an additional 4 ns before entering production runs. For each

nanobody–RBD complex, two independent production simulations of 200 ns each were performed, yielding a total of 400 ns of trajectory per complex.

Supporting Figures

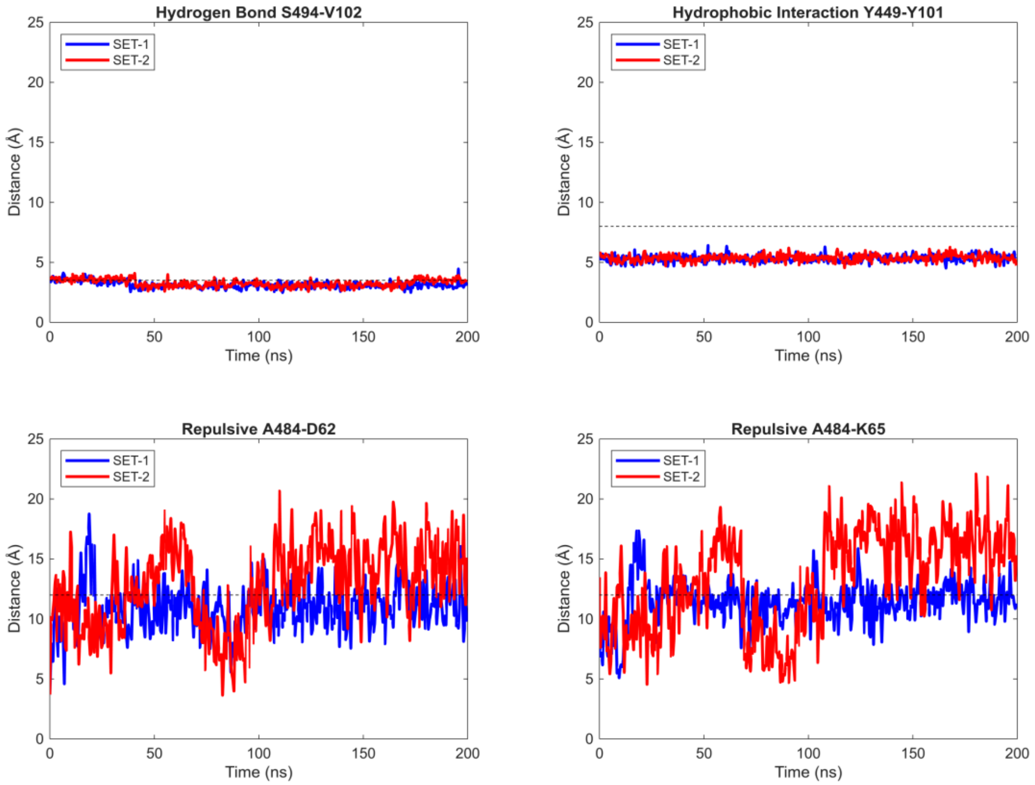

Figure S1. Distance–time traces for correlated and anti-correlated residue pairs between H11-H4 and RBD from Omicron-variant simulations. Distance trajectories are shown for residue pairs forming the S494–V102 hydrogen bond, the Y449-Y101 hydrophobic interaction, and the A484–R62 and A484-K65 hydrophobic–charged repulsive interaction. The dashed line indicates the distance cutoff threshold applied to define the presence of each interaction. Salt bridge formations between H11-H4 and Omicron RBD were not observed in the simulations.

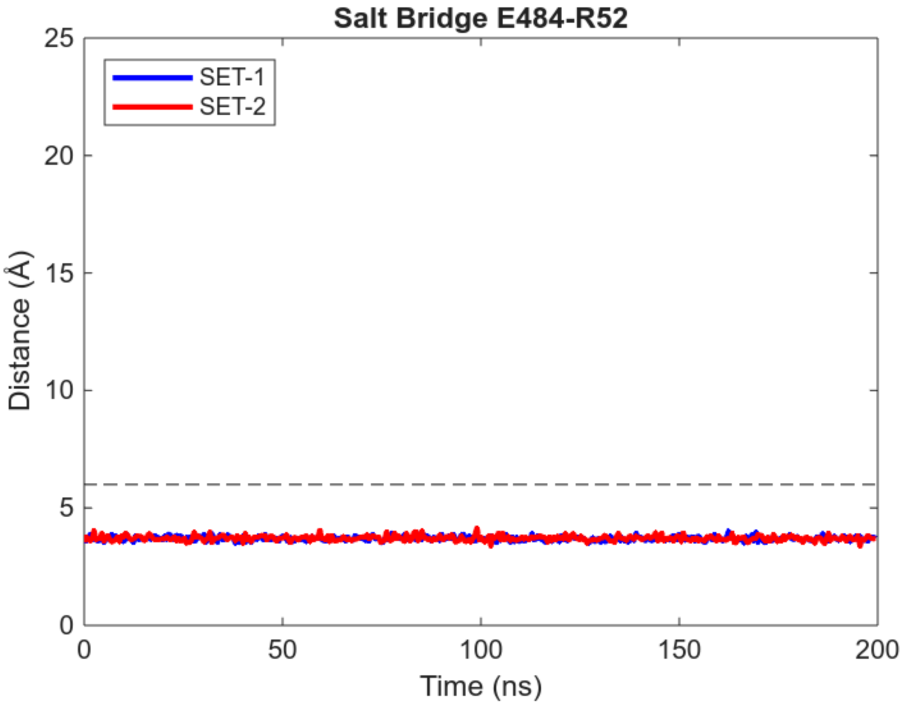

Figure S2. Distance–time traces for the salt-bridge-forming pair between H11–H4 residue R52 and WT RBD residue E484 during the simulations. The dashed line indicates the distance cutoff threshold applied to define the presence of the salt bridge.

References

1 Golcuk, M., Hacisuleyman, A., Erman, B., Yildiz, A. & Gur, M. Binding Mechanism of Neutralizing Nanobodies Targeting SARS-CoV-2 Spike Glycoprotein. *J Chem Inf Model* **61**, 5152-5160 (2021). https://doi.org/10.1021/acs.jcim.1c00695

2 Golcuk, M. *et al.* SARS-CoV-2 Delta Variant Decreases Nanobody Binding and ACE2 Blocking Effectivity. *Journal of Chemical Information and Modeling* **62**, 2490-2498 (2022). https://doi.org/10.1021/acs.jcim.1c01523

3 Huo, J. *et al.* Neutralizing nanobodies bind SARS-CoV-2 spike RBD and block interaction with ACE2. *Nature Structural & Molecular Biology* **27**, 846-854 (2020). https://doi.org/10.1038/s41594-020-0469-6

4 Humphrey, W., Dalke, A. & Schulten, K. VMD: Visual molecular dynamics. *Journal of Molecular Graphics* **14**, 33-38 (1996). https://doi.org/https://doi.org/10.1016/0263-7855(96)00018-5

5 Phillips, J. C. *et al.* Scalable molecular dynamics on CPU and GPU architectures with NAMD. *The Journal of Chemical Physics* **153** (2020). https://doi.org/10.1063/5.0014475

6 Huang, J. & MacKerell Jr, A. D. CHARMM36 all-atom additive protein force field: Validation based on comparison to NMR data. *Journal of Computational Chemistry* **34**, 2135-2145 (2013). https://doi.org/https://doi.org/10.1002/jcc.23354
